# Structure Property Relationship of Micellar Waterborne Poly(Urethane-Urea): Tunable Mechanical Properties and Controlled Release Profiles with Amphiphilic Triblock Copolymers

**DOI:** 10.1021/acs.langmuir.3c00921

**Published:** 2023-07-11

**Authors:** Shu-Yi Chen, Ida Kokalari, Steven R. Parnell, Gregory N. Smith, Bing-Hong Zeng, Tun-Fun Way, Fu-Sheng Chuang, Alina Y Rwei

**Affiliations:** † Department of Chemical Engineering, Delft University of Technology, 2629 HZ Delft, The Netherlands; ‡ Institute of Organic and Polymeric Materials, National Taipei University of Technology, 10608 Taipei, Taiwan; § Research and Development Center for Smart Textile Technology, National Taipei University of Technology, 10608 Taipei, Taiwan; ∥ Department of Radiation Science and Technology, Delft University of Technology, 2629 HZ Delft, The Netherlands; ⊥ ISIS Neutron and Muon Source, Oxfordshire OX11 0QX, U.K.; # Department of Fashion and Design, Lee-Ming Institute of Technology, No. 22, Sec. 3, Tai-Lin Rd., Taishan Dist., New Taipei City 243, Taiwan

## Abstract

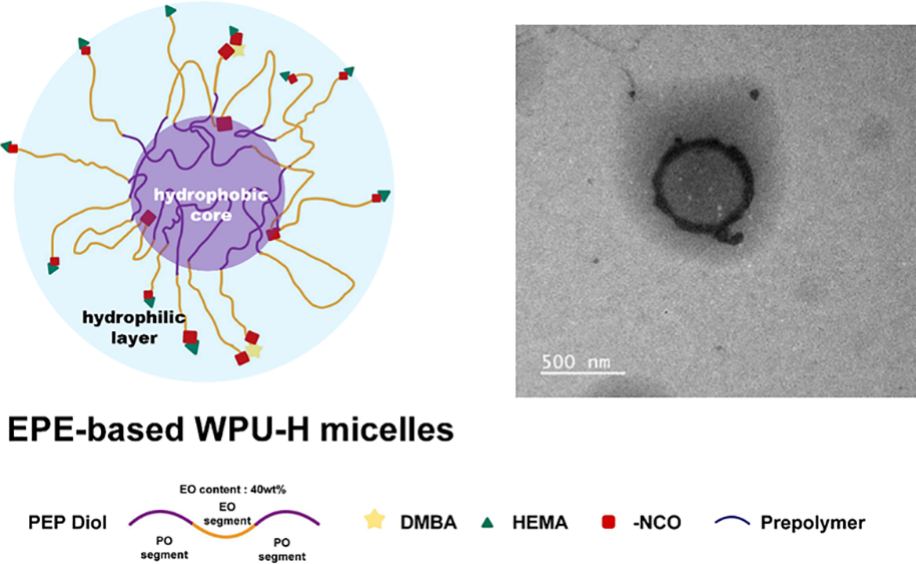

Waterborne polyurethane (WPU) has attracted significant interest as a promising alternative to solvent-based polyurethane (SPU) due to its positive impact on safety and sustainability. However, significant limitations of WPU, such as its weaker mechanical strength, limit its ability to replace SPU. Triblock amphiphilic diols are promising materials to enhance the performance of WPU due to their well-defined hydrophobic–hydrophilic structures. Yet, our understanding of the relationship between the hydrophobic–hydrophilic arrangements of triblock amphiphilic diols and the physical properties of WPU remains limited. In this study, we show that by controlling the micellar structure of WPU in aqueous solution via the introduction of triblock amphiphilic diols, the postcuring efficiency and the resulting mechanical strength of WPU can be significantly enhanced. Small-angle neutron scattering confirmed the microstructure and spatial distribution of hydrophilic and hydrophobic segments in the engineered WPU micelles. In addition, we show that the control of the WPU micellar structure through triblock amphiphilic diols renders WPU attractive in the applications of controlled release, such as drug delivery. Here, curcumin was used as a model hydrophobic drug, and the drug release behavior from WPU-micellar-based drug delivery systems was characterized. It was found that curcumin-loaded WPU drug delivery systems were highly biocompatible and exhibited antibacterial properties in vitro. Furthermore, the sustained release profile of the drug was found to be dependent on the structure of the triblock amphiphilic diols, suggesting the possibility of controlling the drug release profile via the selection of triblock amphiphilic diols. This work shows that by shedding light on the structure–property relationship of triblock amphiphilic diol-containing WPU micelles, we may enhance the applicability of WPU systems and move closer to realizing their promising potential in real-life applications.

## Introduction

Polyurethane (PU) has been widely applied in industrial applications, including coatings,^1−3^ laminates,^4−6^ and biomedical applications such as drug delivery,^7^ due to its high versatility and biocompatibility. This is evidenced by its strong global demand, estimated to be over 20 million tons in 2022.^8^ However, as PU is a water-insoluble polymer, and many organic monomers and oligomers required as PU building blocks are hydrophobic, most PU products are processed with organic solvents, producing toxic and harmful waste products into the environment.^9^ With the awareness of sustainability and the environmental concerns associated with organic-solvent-based processes, waterborne poly(urethane-urea) (WPU) has attracted interest in the past decade due to its environmental sustainability upon the replacement of organic solvents with water during the processing of PU.^10,11^

WPU synthetic processes overcome the limitations of water solubility by the addition of surfactants to form micellar structures in an aqueous solution. However, despite its advantage in reducing environmental concerns, WPU has shown a critical lack of mechanical strength when compared with PU products from traditional synthetic processes,^12−14^ significantly limiting the applications of this sustainable alternative.^15,16^ More specifically, PU is widely applied in coating techniques, forming thin films on various substrates. Yet, WPU exhibits weaker mechanical strength, especially in the form of thin films, when compared with organic solvent-based PU.^17^ This weaker mechanical strength of WPU, when compared with traditional PU, is tightly linked to the microscopic polymeric structures of the resulting films. In organic-solvent-based polyurethane solutions, the polymer chains are extended. When the solvent is vaporized, the polymer chains entangle extensively, enabling strong mechanical properties upon film formation. In WPU, the polymer is dispersed in water with the assistance of hydrophilic groups in the polymer chain, forming self-assembled micelles. Such micelles hinder interpolymer interactions, thereby inhibiting chain entanglement, leading to weaker mechanical properties.^18^ In relationship with chain entanglement, which requires the polymer to reach above a certain molecular weight and enables stronger hydrogen bonds,^19,20^ the molecular weight of WPU is usually lower than that of solvent-based PU since its chain extension process is initiated after the formation of emulsions, leading to low chain extension efficiencies.^21^

These key physical properties of WPU are highly dependent on the microscopic micellar structures of WPU, which are determined by (1) the reaction between isocyanate and polyol/amine and the competing reaction between isocyanate and water during WPU synthesis and (2) micellar coagulation during film formation. The synthesis process determines the molecular weight, as well as the resulting physical properties of WPU.^22,23^ Amphiphilic triblock polyols with the designated hydrophilic–hydrophobic–hydrophilic arrangements are promising materials for the promotion of chain-extended reactions due to their ability to control the self-assembled structure of WPU micelles during synthesis.^24^ These amphiphilic triblock polyols facilitate the reaction of prepolymers with diamine in the aqueous phase, leading to the formation of WPUs with higher molecular weights.

The coagulation of WPU micelles during film formation is another factor that determines the physical properties of the WPU films. During film formation, interpolymer interactions^12,25−27^ mainly based on hydrogen bonds enable the stacking of WPU micelles to form thin films.^28,29^ However, these weak interpolymer hydrogen bonds result in a low mechanical strength when compared with the thin films formed by entanglements as in organic-solvent-based PU. It was previously proposed that end-capping WPU with reactive groups^30,31^ and adding external cross-linkers,^32,33^ e.g., acrylate^34−36^ and silanol,^37−39^ could increase the cross-link density between stacked micelles and enhance the mechanical strength of WPU films. Yet, not only was the contact area between the WPU micelles limited, leading to weak intermicellar interactions, but the residual water at the polyurethane interface was also found to reduce its adhesion strength;^40,41^ these factors resulted in the lack of mechanical strength of WPU-based films. Gaining insight into the structure–property relationship of WPU may enable control of the self-assembled structure of WPU micelles to overcome these limitations and enhance the utility of WPU for real-life applications.

This paper reports a structure–property investigation of WPUs that contain triblock amphiphilic diols as PU’s soft segment to effectively control self-assembled WPU micellar structures and enhance the mechanical performance of WPU. We hypothesize that triblock amphiphilic diols with hydrophilic tail groups may utilize their hydrophilic segments to pull reactive functional groups toward the micellar surface, thereby promoting the cross-linking efficiency and the associated mechanical performance. In this work, we developed amphiphilic WPUs that contain poly(propylene oxide)-*b*-poly(ethylene oxide)-*b*-poly(propylene oxide) triblock diols (PEP) and poly(ethylene oxide)-*b*-poly(propylene oxide)-*b*-poly(ethylene oxide) triblock diols (EPE) to investigate the effect of the triblock amphiphilic diol structure on the cross-linking density of WPU. The resulting WPU exhibited mechanical properties that were dependent on the chemical and physical structures of WPU micelles. Formulations showing enhanced mechanical strength that are promising for real-life applications are also presented.

The ability to control micellar structures via amphiphilic diols may be utilized in a wide range of WPU applications. One of the promising applications of micellar-structured WPU is in controlled release, e.g., drug delivery. By investigating the self-assembled WPU structures and the drug–polymer interactions via the hydrophilic and hydrophobic arrangements of amphiphilic diols, we may determine the design principles that determine the temporal drug release profile from WPU-based drug delivery systems. In this paper, we demonstrate the application of amphiphilic WPU as a drug delivery carrier loaded with curcumin in the application of antibacterial wound dressing. Curcumin is a natural compound extracted from the root of *Curcuma longa* with promising antibacterial^42−44^ and antitumor^42,45−47,48,49^ properties. However, curcumin exhibits a high degradation rate under alkaline condition in its diketone form and low solubility under acidic condition with its enol form.^50^ These special properties lead to the low bioavailability of curcumin, limiting its therapeutic application.^51,52^ Nonionic surfactants, including PU, have been previously used to stabilize the hydrophobic curcumin in aqueous solution.^51,53−55^ However, the curcumin release rate from these systems is high and difficult to manipulate.^55−57^ Furthermore, an understanding of the relationship between PU-based micellar structures and drug release kinetics remains limited. In this research, we report a curcumin-sustained release system based on a triblock amphiphilic diol consisting of WPU. The relationship between these WPU micellar structures and their corresponding drug release kinetics was investigated. The structure-dependent drug release behavior revealed a technique to manipulate the drug release rate by the design of the amphiphilic diol. The resulting curcumin-loaded WPU exhibited significant antibacterial effects and biocompatibility.

In summary, this paper reports on the structure–property investigation of WPU systems based on triblock amphiphilic diols. We show that a deeper understanding of the relationship between the hydrophobic–hydrophilic structure and WPU particle formation in aqueous solution enables the development of WPU with the following key attributes: (1) enhanced mechanical properties; (2) efficient drug encapsulation that protects the drug from chemical degradation; (3) tunable drug release profile via the selection of soft segment diols; and (4) effective antibacterial properties upon curcumin encapsulation.

## Experimental Section

### Materials

4,4′-Methylenebis (cyclohexyl isocyanate) (HMDI, H12MDI), 2,2-bis(hydroxymethyl)butanoic acid (DMBA), triethylamine (TEA), acetone (99.5%), Tween-80, ethylenediamine (EDA), polypropylene glycol (PPG, Mn = 2000), 2-hydroxyethyl methacrylate (HEMA), osmium tetroxide (4.0 wt % in water), and dibutyltin dilaurate (DBTDL, 95%) were supplied by Sigma-Aldrich and used without further purification. Poly(propylene oxide)-*b*-poly(ethylene oxide)-*b*-poly(propylene oxide) triblock diols (PEP diols), PEP-25 (EO/PO = 25/75 wt %, Mw = 2200 g mol^–1^) and PEP-35 (EO/PO = 35/65 wt %, Mw = 2000 g mol^–1^), poly(ethylene oxide)-*b*-poly(propylene oxide)-*b*-poly(ethylene oxide) triblock diols (EPE diols), EPE-40 (EO/PO = 40/60 wt %, Mw = 2200 g mol^–1^) and EPE-20 (EO/PO = 20/80 wt %, Mw = 2400 g mol^–1^), and PPG (Mw = 2000 g mol^–1^) were sourced from Enhou Polymer Chemicals, Taiwan. The photoinitiator 2-hydroxy-4′-hydroxyethoxy-2-methylpropiophenone was purchased from Double bond Chemical Ind.

Phosphate-buffered saline (PBS), high-glucose Dulbecco’s modified Eagle's medium (DMEM), tryptic soy broth, and 10% fetal bovine serum (FBS) were supplied by Merck, Germany. Gibco antibiotic–antimycotic was supplied by Thermo Fisher. The six-well Transwell plate (1.2 mm diameter, 0.4 μm pore polyester membrane) was supplied by Corning, USA. The A549 cell line and *Escherichia coli* (ATCC 25922) were purchased from ATCC (American Type Culture Collection). The MTT (3-(4, 5-dimethylthiazolyl-2)-2, 5-diphenyltetrazolium bromide) assay kit (CyQUANT MTT Cell Viability Assay) was supplied by Thermo Fisher.

### Synthesis of WPU with Triblock Amphiphilic Diols as Soft Segment

**Scheme 1:**
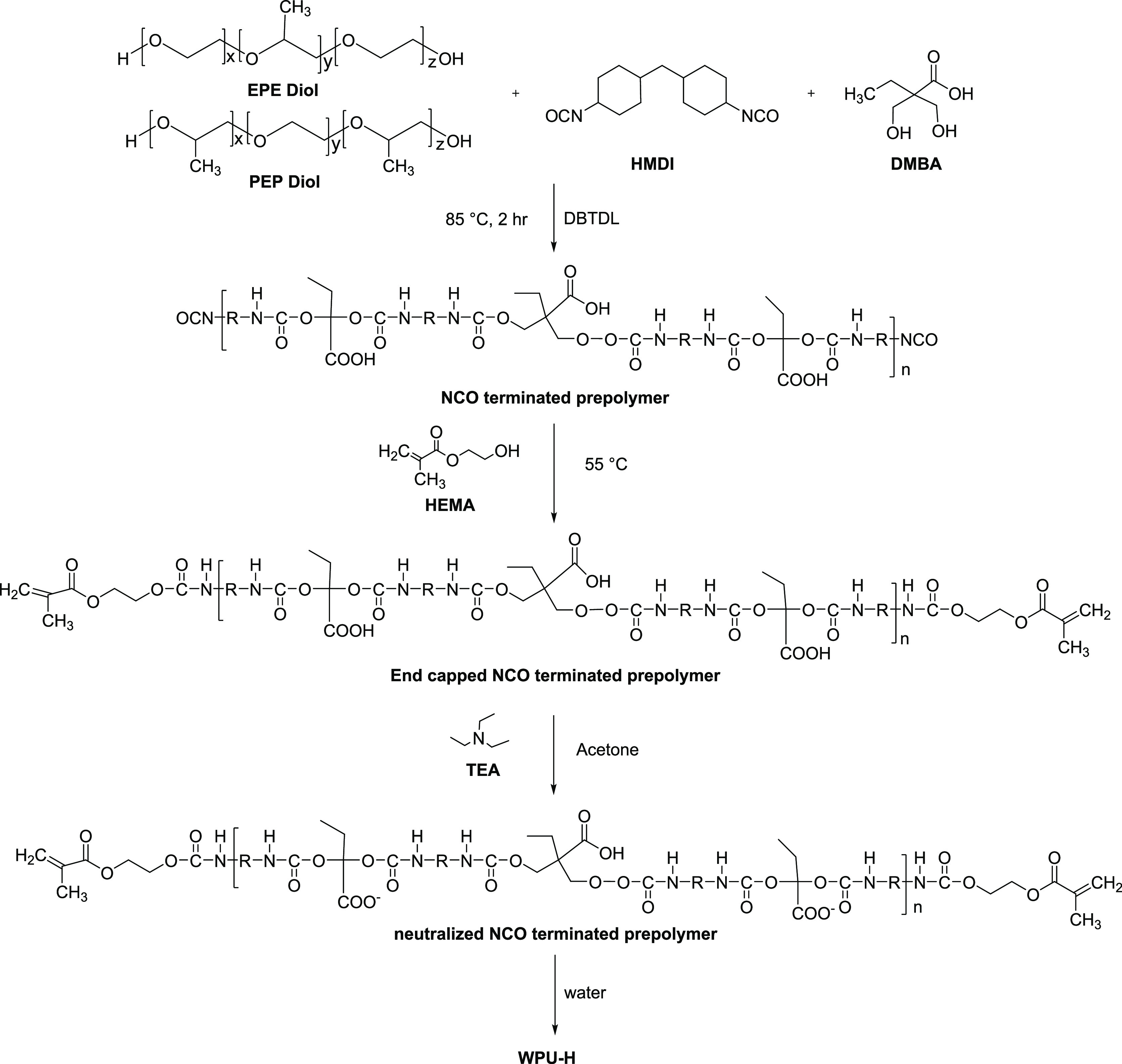
Synthetic Scheme of WPU-H

WPU-H was synthesized by the prepolymer method (Scheme 1) according to the compositions listed in Table S1. First, diols were dehydrated under a vacuum at 100 °C before synthesis. The diols, DMBA, HMDI, and DBTDL were stirred by a mechanical stirrer with Teflon stirring blades (150 rpm) in a round flask at 85 °C under nitrogen until the isocyanate content of the prepolymer, determined by titration, reached the theoretical value. Then, the temperature was decreased to 50 °C, and HEMA was added with stirring, until the isocyanate content reached zero. After the obtained oligomer was cooled to 40 °C, TEA dissolved in acetone was added to neutralize the oligomer for 30 min. Finally, the emulsification process proceeded by adding water into the oligomer through rigid stirring at room temperature. After the emulsification process, the residual organic solvent in WPU-H was recovered by a rotary evaporator under 80 mmHg with a 40 °C water bath. The samples were named after their diol type, the PEO content in the diol, and the content of HEMA. PPG-H7 was synthesized as a control sample without PEO.

**Scheme 2:**
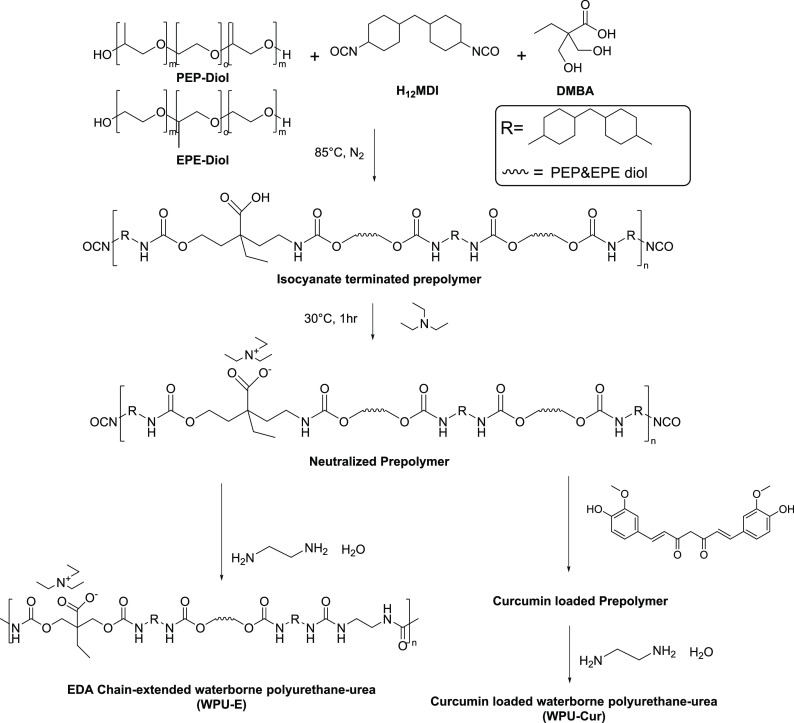
Synthetic Scheme of WPU-E and WPU-Cur

WPU-E and WPU-Cur were also synthesized by the prepolymer method (Scheme 2) and the same synthesis protocol of prepolymer as WPU-H according to the composition listed in Tables S1 and S2. After neutralization, curcumin was dissolved in acetone (20 mL) and mixed with the prepolymer before the emulsification of WPU-Cur. WPU-E was prepared without curcumin. Finally, the emulsification and chain-extending processes were proceeded by adding EDA aqueous solution into the oligomer through rigid stirring at room temperature. WPU-E and WPU-Cur were synthesized after solvent removal and named after their diol type and PEO content in the diol.

### Preparation of Triblock Amphiphilic Waterborne Polyurethane–Urea Films

Before casting, WPU-H was mixed with 1 wt % of 2-hydroxy-4′-hydroxyethoxy-2-methylpropiophenone. WPU-E, WPU-H, and WPU-Cur films were cast on silicone rubber molds and dried at room temperature for 3 days, followed by incubation at 60 °C for 12 h to obtain a dry film with a thickness of 0.3–0.5 mm. WPU-H films were cured by irradiating with a UV lamp (wavelength: 250–360 nm) under nitrogen for 15 min.

### Polymer Characterization

FTIR spectra were recorded on a PerkinElmer Spotlight 200i spectrometer in attenuated total reflection (ATR) mode. Gel permeation chromatography (GPC) was run by a Malvern GPC system with 270 and 3580 RI detectors and a Shodex GPC KF-805L column. GPC samples were dissolved in 5 mL of DMF (0.02 M LiBr) solution and operated at 25 °C. The particle size distribution and zeta potential were measured by dynamic light scattering (DLS) and electrophoretic light scattering (ELS) to determine the stability of WPU by using a NanoBrook 90Plus PALS system with 0.1 wt % WPU. Thermogravimetric analysis (TGA) was run on a Hitachi STA7200 system at a heating rate of 10 °C/min, from 50 to 600 °C, under nitrogen. Differential scanning calorimetry (DSC) was conducted on a Hitachi DSC 7000X system with three stages, 25–150 °C, 150 to −150 °C, and −150 to 150 °C, at a heating/cooling rate of 5 °C/min to obtain the phase transition temperatures of WPU. Dynamic mechanic analysis (DMA) was conducted at a frequency of 1 Hz, amplitude of 10 μm, and temperature of −100 to 150 °C, 5 °C/min on a Seiko DMS 6100 system with the tensile mode; the sample size was 20 × 10 × 0.2 mm. The stress–strain curve (S–S curve) was recorded by a ComeTech QC-508M2F tensile testing machine at a testing speed of 500 mm/min. The specimen shape and size were according to the ASTM D638 standard.

### Determination of the Cross-Link Degree of WPU-H Film Samples

For the calculation of the molecular weight between the cross-link points (*M*_c_), the storage modulus (*E*′) at the rubbery plateau was measured by DMA and calculated according to the classical theory of elasticity^58^ as follows:
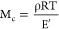
1where ρ is the density of the elastomer, *R* is the gas constant, *E*′ is the storage modulus at the rubbery plateau, and *T* is the absolute temperature.

### Curcumin Loading Efficiency of WPU-Cur and Stability of Curcumin in PBS

The loading efficiency of curcumin in WPU-Cur was measured using a UV–vis spectrophotometer (Hitachi UH5300 system), and the concentration was calculated according to the calibration curve, in the concentration range of 2–10 μg/mL, at λ = 435 nm. The WPU-Cur film samples (3 mg) were dissolved in *N*-methyl-2-pyrrolidone (NMP) (20 mL) prior to the measurements. The loading efficiency was calculated by the ratio between the theoretical and measured curcumin concentrations. As a control sample, the curcumin PBS solution (10 μg/mL) was prepared, and the pH value was adjusted to 7.8 for simulating the pH value of WPU-Cur.

### Curcumin Release Test of WPU-Cur and Drug Release Mechanism Model Analysis

To investigate the curcumin-releasing behavior of WPU-Cur, the sample films (15–20 mg) were incubated in PBS (pH 7.4, with 0.5 wt % Tween-80) at 37.5 °C. At specified time points, the samples were centrifuged at 3000 rpm for 15 min to separate the disintegrated WPU. To measure the curcumin absorbance (at 427 nm), the supernatant was mixed with methanol (1:1 v.v.) and analyzed using a UV–vis spectrophotometer (Hitachi UH5300 system). The concentration of curcumin was further calculated according to the calibration curve.

The accumulated curcumin release curve was fitted to the Gallagher–Corrigan model.^59,60^ The Gallagher–Corrigan model described two stages of drug releasing, bursting and decomposing. The bursting stage, which is related to the free drug and to the drug that adheres on the surface of the polymer, is characterized by a relatively faster releasing rate. The second stage, named the decomposition stage, was triggered by the decomposition or disintegration of the polymer. The Gallagher–Corrigan model equation is shown as follows
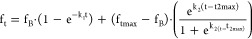
2where *f*_t_ is the fraction of the accumulated released drug, *f*_B_ is the fraction of the released drug in the bursting stage, *k*_1_ is the releasing constant of the bursting stage, *k*_2_ is the releasing constant of the decomposing stage, *t* is the released time, *t*_2max_ is the time of the maximum releasing rate in the decomposing stage, and *f*_tmax_ is the fraction of the maximum released drug.

### Disintegration Test of the WPU-Cur Film

To investigate the disintegration of WPU-Cur during the drug release process, the WPU-Cur films were incubated in PBS buffer (10 mM, pH 7.4, 0.5 wt % Tween-80) at 37.5 °C. The samples were then dried in an oven (60 °C, 2 h) and weighed, and finally, the molecular weight was determined by GPC. The weight loss during the incubation was calculated by the following equation
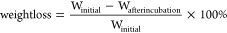
3where *W*_initial_ is the weight of the sample before incubation, and *W*_after incubation_ is the weight of the dried sample after incubation in PBS.

### Swelling Test of the WPU-Cur Film

The WPU-Cur samples were incubated in PBS buffer (10 mM, pH 7.4, 0.5 wt % Tween-80) at 37.5 °C. At the desired time point, the samples were dried by wiping the residual buffer on the surface of the sample film and weighed. The swelling degree was calculated by the following equation:
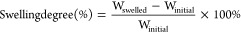
4where *W*_initial_ is the weight of the sample before incubation, and *W*_swelled_ is the weight of the sample after incubation in PBS.

### Cytotoxicity Test of the WPU-Cur Film

The effect of the WPU-Cur film on the human A549 lung adenocarcinoma cell viability was investigated using the colorimetric MTT assay. The A549 cell line was cultured in high-glucose DMEM supplemented with 10% FBS and 1% Gibco antibiotic–antimycotic in a humidified incubator at 37 °C in a 5% CO_2_ atmosphere. The WPU film samples were prepared from the main batch at 5 mg size and sterilized using UV irradiation for 30 min on each side.

On day 1, A549 cells were seeded into the basal compartment of a six-well Transwell plate at a density of 2 × 10^4^ cells/well in a volume of 1 mL of fresh cell culture medium and incubated for 24 h. On day 2, the A549 cells were exposed to WPU film samples by placing them on the upper compartment of the Transwell plate, followed by 24 or 48 h of incubation. After the exposure, the medium was refreshed, and 50 μL of 12 mM MTT solution was added to each well. The samples were incubated for 4 h. After incubation, the medium was replaced with 500 μL of SDS (sodium dodecyl sulfate)–HCl solution (10% in 0.01 M HCl) and incubated for 4 h. Finally, the absorbance at 570 nm was recorded for each sample by a microplate reader (Multiskan FC, Thermo fisher). Results are the average of two independent experiments.

### Antibacterial Test of the WPU-Cur Film

The antibacterial property of the WPU-Cur film was determined by the modified ASTM E2149 standard^61^ under dynamic contact conditions. The Gram-negative bacteria *Escherichia coli* (ATCC 25922) was used as a model organism. Briefly, WPU-Cur and WPU-E (control) samples (2.0 g each) were cut into small pieces and transferred to a 250 mL flask containing 50 mL of the bacterial suspension. The bacterial suspension was prepared in a nutrient broth (tryptic soy broth) and then diluted with 0.3 mM PBS (pH = 6.8) to obtain a final concentration of 1.5–5.5 × 10^5^ colony-forming units (CFU)/mL. All the flasks were incubated and shaken for 1 h at 37 °C. After a series of dilutions, 1 mL of the diluted suspension was spread on plate count agar. The plates were incubated at 37 °C for 24 h, and the CFUs were counted. The average values of the duplicates were, after multiplying by the dilution factor, converted into CFU/mL in the flasks. The antimicrobial activity was calculated by the following equation
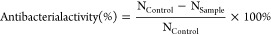
5where *N*_Control_ and *N*_Sample_ are the CFU/mL of control (WPU-E) and curcumin-loaded sample (WPU-Cur), respectively.

### Small-Angle Neutron Scattering Measurement of WPU

Small-angle neutron scattering (SANS) measurements were performed on the LARMOR instrument at the ISIS Neutron and Muon Source (Rutherford Appleton Laboratory, United Kingdom).^62^ LARMOR is a fixed-configuration time-of-flight, pinhole SANS instrument with a sample-to-detector distance of 4 m. The usable wavelength (λ) range on T2 at ISIS is 0.9 < λ < 13.5 Å, which gives a maximum *q* range of 0.005 < *Q* < 0.7 Å^–1^, where *Q* is the magnitude of the momentum transfer vector (*Q* = (4 π sin(θ)/λ). Data were converted from raw data to reduced data of scattering intensity as a function of *Q* by correcting for detector efficiency and sample transmission using the instrument-specific software Mantid.^63,64^ The raw data were placed on an absolute scale (cm^–1^) by measuring the scattering of a mixture of hydrogenous and deuterated polystyrene with the known radius of gyration and scattering cross section^65^

The WPU samples were dried and dissolved in acetone. Then, the solution was emulsified in D_2_O under vigorous stirring. Acetone was removed by a rotary evaporator after emulsification. H_2_O was used to prepare samples with different D_2_O volume fractions. The samples were loaded in rectangular quartz cells with a thickness of 1 mm and were illuminated by a 6 × 8 mm^2^ beam. All of the data were corrected for background and solvent scattering by subtraction using a blank (pure D_2_O), processed the same way as the samples.

## Results and Discussion

### FT-IR Analysis of WPU with Triblock Amphiphilic Diols as Soft Segment

**Figure 1:**
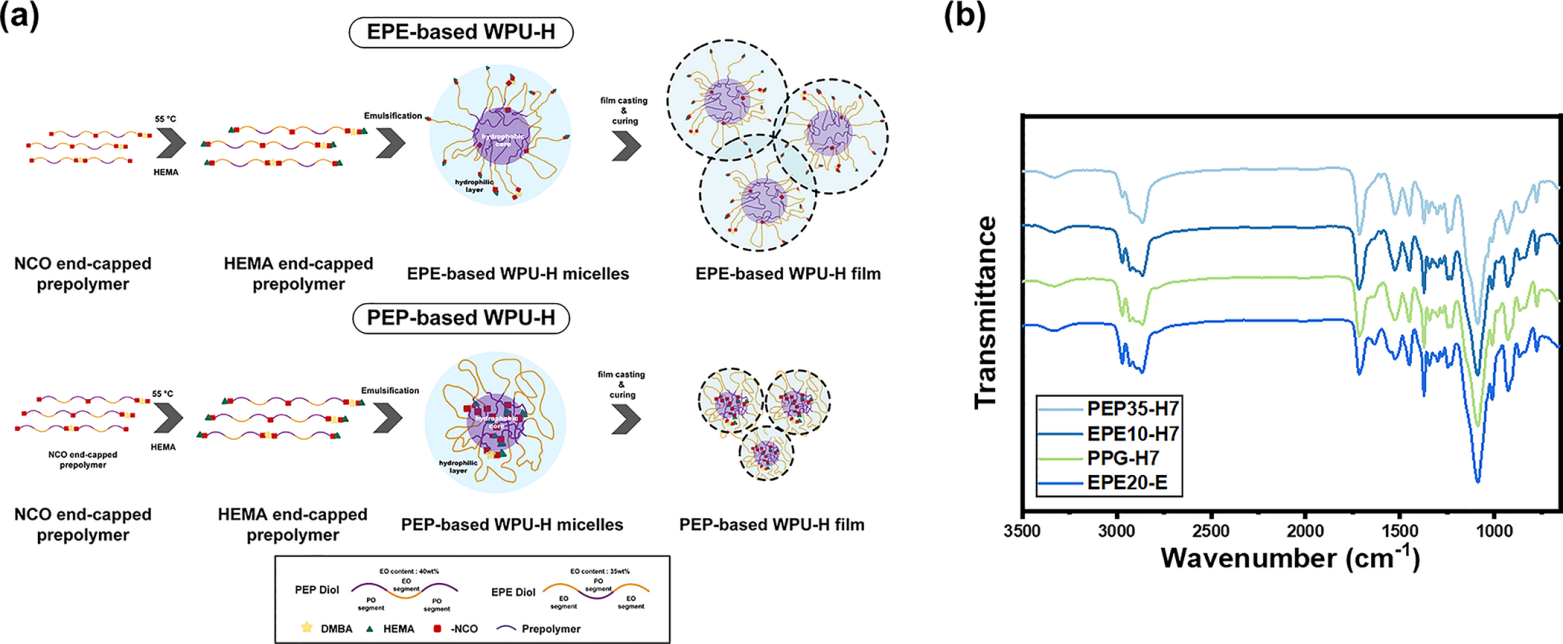
Synthesis of WPU polymers with varied arrangements of hydrophobic segments: (a) illustration of WPU with amphiphilic segments, which scattered the acrylate groups on the surface of micelles; (b) FT-IR spectra of WPU with varied diols (e.g., EPE diols, PEP diols, and PPG)

WPU polymers based on triblock amphiphilic diols with varied distributions of hydrophilic (PEO) and hydrophobic (PPO) segments were synthesized to investigate the self-assembly and micellar properties of triblock-amphiphilic-diol-based WPU. The synthesis steps are shown in Schemes 1 and 2 (detailed in Experimental Section), and the hypothesized micelle assembly process is illustrated in Figure 1a. The FT-IR spectra of the WPU prepolymer revealed the presence of characteristic peaks corresponding to the isocyanate groups in WPU (Figure S1), while such peaks were absent in EPE20-H7, suggesting the successful polymerization of polyurethane-urea in the WPU sample. Figure 1b shows the FT-IR spectra of 2-hydroxyethyl methacrylate (HEMA) end-capped triblock amphiphilic waterborne polyurethane-urea (WPU-H) and ethylenediamine (EDA) chain-extended polyurethane-urea (WPU-E). The characteristic peaks of urethane and urea, located at 3400–3460 cm^–1^ (N–H stretching vibration), 1670–1730 cm^–1^ (C=O stretching vibration), 2980 cm^–1^ (−CH stretching), and 1223–1100 cm^–1^ (C–O–C stretching), indicated the successful synthesis of polyurethane-urea. Furthermore, there was no peak at 2270 cm^–1^, indicating the reaction of the isocyanate groups. These results demonstrate the successful synthesis of WPU based on poly(propylene oxide)-*b*-poly(ethylene oxide)-*b*-poly(propylene oxide) triblock diols (PEP diol) and poly(ethylene oxide)-*b*-poly(propylene oxide)-*b*-poly(ethylene oxide) triblock diols (EPE diol).

### Physical Properties of WPU Polymers with Varied Arrangements of Hydrophobic Segments Controlled via Triblock Amphiphilic Diols

Table S3 summarizes the molecular weight and glass-transition temperature of the WPU polymers synthesized in this study. WPU synthesized from EPE diols showed higher molecular weights when compared with WPU from PEP diols with the same HEMA content (Table S3), demonstrating a higher chain-extending efficiency. This suggests that the hydrophilic poly(ethylene oxide) tail of EPE diol assisted the hydrophobic isocyanate groups of urethane oligomers to react with the aqueous phase chain-extender, EDA. In the PEP system, however, the hydrophobic group tends to be packed in the core of micelles due to the hydrophobic poly(propylene oxide),^66^ shielding the end groups from the aqueous phase and their access to the cross-linker EDA, thereby hindering further chain extensions (Figure 1a). These results show that the distribution of hydrophilic and hydrophobic segments in the triblock amphiphilic diols plays a significant role in the surface distribution of functional groups and thereby the resulting chain extension efficiency of WPU.

The effect of end-capped groups was also investigated (Table S3). The molecular weight of WPU-H was lower than that of WPU-E because the oligomer formed during the synthesis of WPU-H was end-capped with the hydroxyl groups of HEMA, while the oligomers in WPU-E synthesis reacted with diamines that could be further reacted with oligomers for chain-extension.

The glass-transition temperature (*T*_g_) of WPU samples was measured by DSC. *T*_g_ of WPU ranged between −60.3 and −54.1 °C (Table S3), showing that the samples are in the rubbery state at the temperatures related to this study. These results are expected and supported by previous studies.^67,68^

### Micellar Properties of Self-Assembled WPU in Aqueous Solution

**Figure 2:**
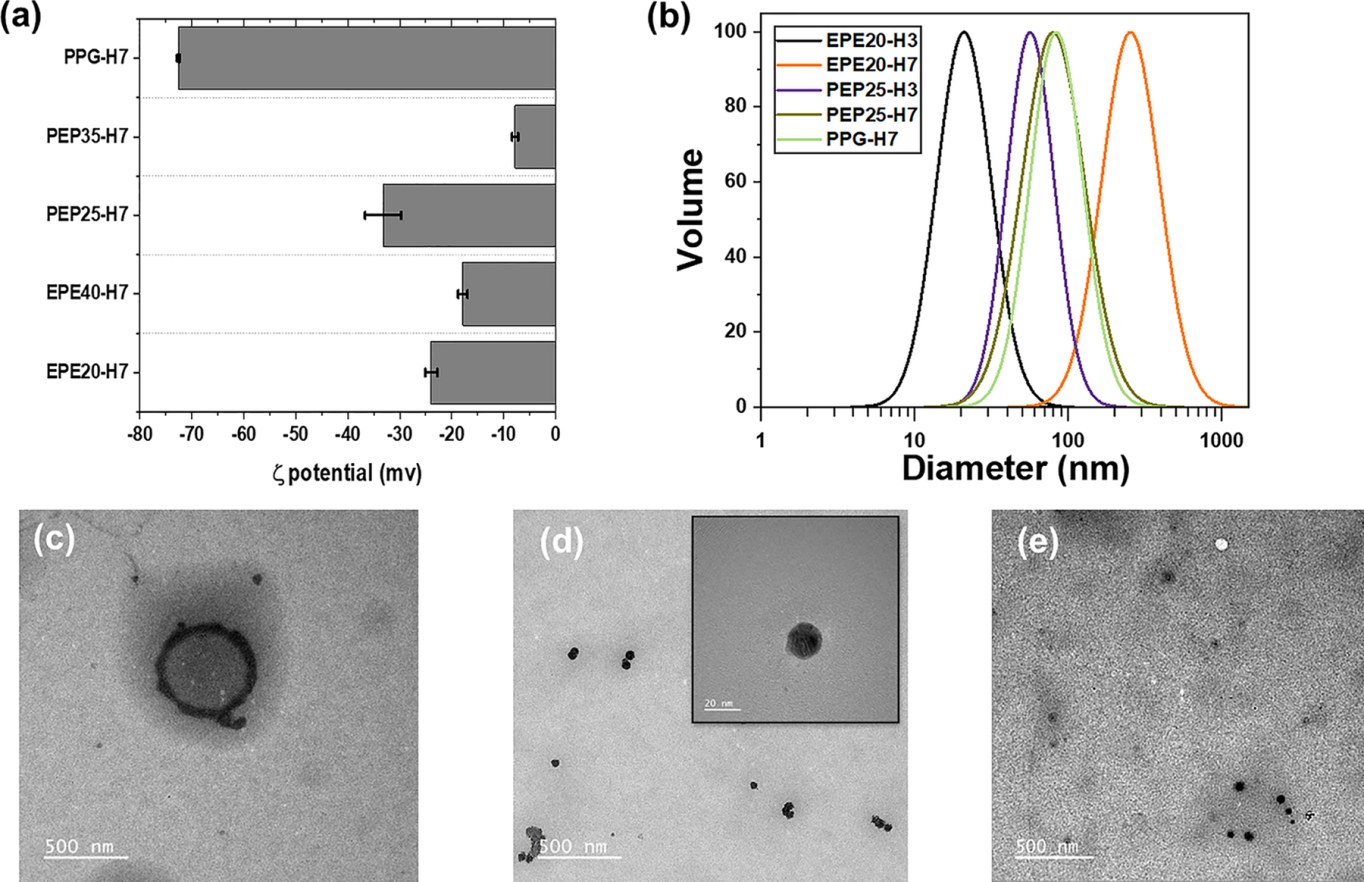
Structure property relationship of the WPU polymeric structure and the self-assembled micelles. (a) ELS for the ζ potential of WPU-H. The EO content is detailed in Experimental Section. (b) DLS for the particle size distribution of WPU-H. The TEM images of EPE20-H7 (c), PEP25-H7 (d), and EPE20-H3 (e) were stained by OsO4, and HEMA is shown in darker shade.

Figure 2a,b presents the ζ potential (determined by electrophoretic light scattering, Figures 2a, S2, and Table S3) and the particle size (determined by dynamic light scattering, Figure 2b) of the WPU-based micelles.

#### Zeta Potential

WPU showed a decrease in the absolute value of ζ potential with an increasing hydrophilic PEO content (Figure 2a; the EO content of each formulation is detailed in Experimental Section). This behavior is expected and consistent with our previous study,^69^ as the hydrophilic EO segments could appear at the surface of micelles, thereby hindering the exposure of the carboxylic acid groups of DMBA at the surface. Compared with the EO-containing WPU samples, the PPG-containing polymer (PPG-H7) exhibited a higher absolute ζ potential value, since PPG is hydrophobic, and its stabilization in aqueous solution relies only on the carboxylic groups instead of the EO segment. These results confirm that the surface distribution of functional groups is sensitive to the distribution of hydrophilic and hydrophobic segments of the triblock amphiphilic diols, which impacts the chain-extending efficiency and cross-linking efficiency, as well as the corresponding molecular weight and physical properties, of WPU.

#### Particle Size and Morphology Characterization

Figure 2b shows the particle size distribution of the WPU-H micelles characterized by DLS. EPE20-H3 is smaller than that of PEP25-H3 in DLS analysis. This is potentially because EPE-based WPU contains hydrophilic PEO tails that effectively shield the hydrophobic core, thereby decreasing the interfacial energy, resulting in a lower aggregation number per particle and, therefore, a smaller particle size.^70^

The particle size of EPE-containing samples exhibited a strong dependence on the HEMA content, with EPE20-H3 at 21.03 ± 0.69 nm and EPE20-H7 at 257 ± 16 nm. The dependence of HEMA on EPE-containing particles is characterized by a change in particle morphology, as shown by TEM (Figure 2c–e). During the TEM characterization, OsO_4_ was used as a staining agent to visualize the hydrophobic tail groups (HEMA). The TEM image of EPE20-H7 showed that HEMA was distributed on the surface of the particle, exhibiting a vesicle-like structure, while the TEM characterization of EPE20-H3 showed that HEMA was distributed in the core of the particle.^71^ The dependence of the self-assembled EPE-containing WPU structures on the HEMA content is reflected in the dependence of particle morphology on the volume fraction of the hydrophobic and hydrophilic segments. With the dominant hydrophilic volume fraction (as in EPE20-H3), the radius of curvature at the interface between the hydrophobic and hydrophilic segments is small and likely cone-shaped, leading to a micelle formation, while an increase in hydrophobicity (as in EPE20-H7) increases the radius of curvature, therefore likely changing into a cylinder shape, leading to vesicle formation.^72^

Furthermore, the TEM image of EPE20-H7 showed that HEMA was distributed on the surface of the particle, demonstrating that the hydrophobic HEMA segments were pulled near the particle surface by EO segments. This self-assembled structure of EPE20-H7 indicates that intermicellar cross-linking may be promoted during film formation due to the significant surface distribution of HEMA. On the other hand, the TEM image of PEP25-H7 showed that the HEMA segments were distributed in the hydrophobic core, indicating that the hydrophobic PPO tail group inherently limited the access of HEMA to the aqueous interface to minimize the entropy penalty upon contact with aqueous solution, thereby limiting its ability to form intermicellar cross-links. As will be presented in the later section of this study on the application of these particles in the encapsulation of hydrophobic molecules, the distribution of encapsulated hydrophobic molecules in these particles can be affected by the particle morphology.^73−75^

### Dynamic Mechanical Analysis (DMA) of WPU-H

**Figure 3:**
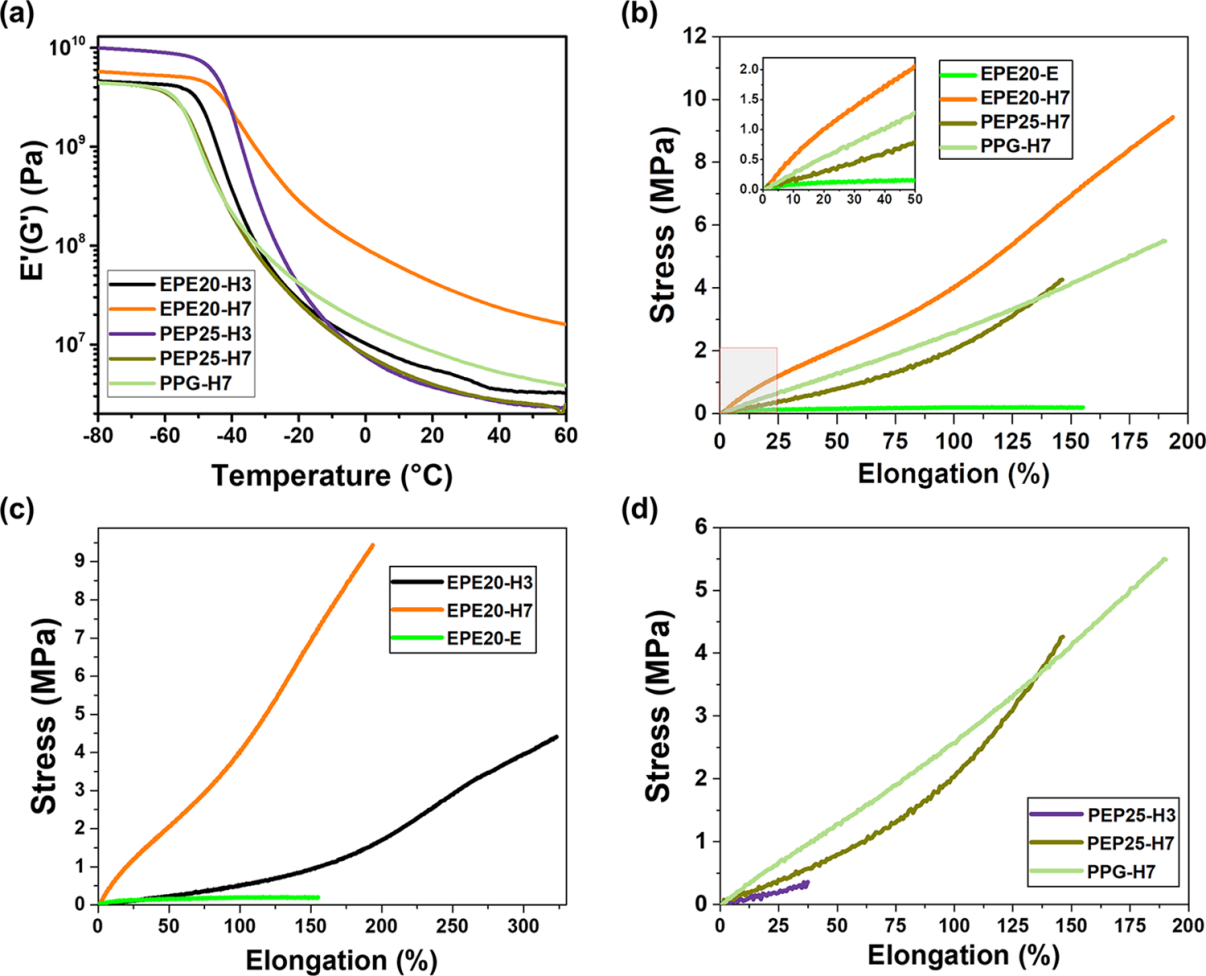
Mechanical properties of WPU-H films. (a) Storage modulus (*E*′) of WPU-H with respect to temperature. *E*′ at the rubbery plateau of samples was used to determine the cross-linking density. (b) Stress–strain curves of PPG-, EPE diol-, and PEP-diol-based WPU-H compared with those of WPU-E. (c) Stress–strain curves of EPE-based WPU-H with varied HEMA contents. (d) Stress–strain curves of PEP-based WPU-H with varied HEMA contents.

The mechanical properties and cross-linking density of WPU-H films were characterized by DMA and further analyzed by the classical rubber theory (Figure 3).^58^Figure 3a demonstrates that WPU-H based on EPE diols exhibited a higher storage modulus at the rubbery plateau region, indicating a higher cross-linking density of these structures, when compared with films based on the PEP and PPG diols. This higher cross-linking efficiency of EPE-based WPU suggests that the surface distribution of HEMA, promoted by the PEO tail groups of EPE, and its corresponding vesicle-like structure promoted intermicellar cross-linking. With a higher HEMA content, EPE20-H7 showed a higher storage modulus than EPE20-H3, suggesting that the acrylate groups in HEMA promoted covalent cross-linking upon photopolymerization. PEP25-H3 showed the lowest storage modulus, possibly because the hydrophobic acrylate groups of HEMA were distributed mainly in the core of the micelles during self-assembly to minimize the enthalpic penalty, thereby decreasing its ability to form intermicellar cross-linking during photopolymerization. Even with increased HEMA content, PEP25-H7 only displayed a slight increase in storage modulus, supporting the hypothesis that surface distribution of HEMA is key to the enhancement of cross-linking density and its associated mechanical strength. Similarly, because of the lack of hydrophilic groups in the soft segment of PPG-H7, the inaccessibility of cross-linking functional groups on the micellar surface led to a low modulus of PPG-H7 films.

### Tensile Test of WPU-E and WPU-H

The mechanical strength of the WPU samples was further quantified by a tensile test. Figure 3b–d presents the resulting stress–strain curves (S–S curves). Figure 3b shows the importance of controlling the surface distribution of HEMA with amphiphilic diols. EPE20-H7 exhibited a high modulus of 0.049 MPa and a breaking stress of 9.43 MPa, while PEP25-H7 showed a lower modulus of 0.029 MPa and a breaking stress of 4.26 MPa. These results indicated that the increase in the intermicellar cross-linking density of EPE-H7 due to the high surface distribution of HEMA upon self-assembly substantially improved the mechanical strength. On the other hand, EPE-based WPU films without the HEMA cross-linking groups, EPE20-E, exhibited a low modulus of 0.0012 MPa and breaking stress of 0.20 MPa due to the lack of intermicellar covalent bonds, demonstrating a weak mechanical strength. As a control sample, that is in the absence of hydrophilic soft segments, PPG-H7 showed a modulus of 0.029 MPa and breaking stress of 5.49 MPa, lower than that of EPE-based, HEMA end-capped WPU. These results indicate that the arrangement of EO segment in the soft segment of WPU, and the corresponding position of HEMA upon self-assembly, plays a critical role in the mechanical properties of WPU films.

Figure 3c,d further demonstrates the importance of the HEMA content in the mechanical properties of WPU films. Figure 3c shows that the stress and elongation at break were enhanced greatly upon the introduction of acrylate groups to the amphiphilic EPE-based WPU system; this improvement underscores the potential utility of the modified WPU in various applications. Furthermore, in Figure 3d, PEP25-H7 shows a drastic improvement in both stress and elongation at break compared to PEP25-H3. These results show that the HEMA content is a crucial factor for the cross-linking density and the mechanical strength of WPU films.

### Characterization of the WPU-Cur Polymer

**Figure 4:**
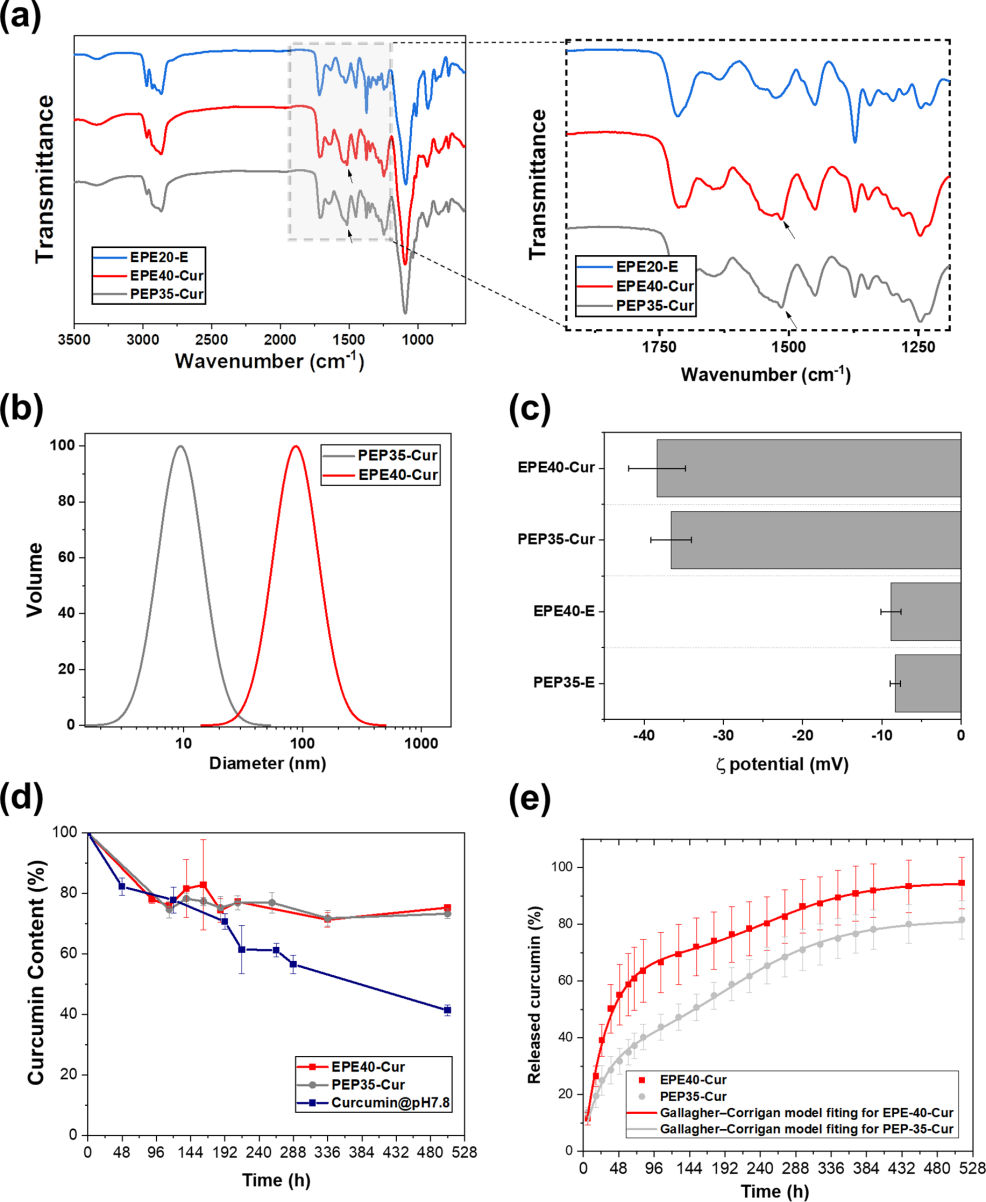
Characterization of WPU-Cur. (a) FTIR spectra of WPU-Cur and WPU-E. The peak at 1509 cm^–1^ is related to curcumin in WPU-Cur. (b) Particle size distribution of WPU-Cur. (c) ζ potential of WPU-Cur. (d) Loading efficiency of WPU-Cur, and the curcumin concentration versus storage time, at room temperature, pH = 7.8. (e) Accumulative released curcumin curve of WPU-Cur and the fitted curve of EPE40-Cur and PEP35-Cur according to the Gallagher–Corrigan model.

In addition to the enhanced mechanical strength, the tunable particle structure based on controlling the amphiphilic polyol content renders it effective in controlled-release applications, such as drug delivery. Curcumin is a promising natural antibacterial and anti-inflammatory agent, showing high biocompatibility in various drug delivery systems.^76,77^ However, its instability and low solubility in aqueous solutions have limited its medical applications. Here, we demonstrate an example of utilizing amphiphilic polyol-based WPU films for the delivery of curcumin in antibacterial therapeutic applications. Figure 4a demonstrates the FT-IR characteristics of the WPU-Cur polymers. WPU-E showed the typical polyurethane-urea spectra, as described in the previous section. Curcumin-loaded triblock amphiphilic waterborne polyurethane-urea (WPU-Cur) showed more intense peaks related to the C=O and C=C vibrations of the curcumin structure at 1509 cm^–1^. The molecular weights of the WPU-Cur samples were comparable to that of the noncurcumin-loaded samples (Table S4), as expected.

### Characterization of WPU-Cur Particles in Aqueous Solution

Figure 4b,c characterizes the size and zeta potential of the self-assembled WPU-Cur particles. EPE40-Cur formed a particle size of 86 ± 2 nm and PEP35-Cur 9 ± 1 nm. This difference may stem from the particle morphology based on the EO arrangements in these amphiphilic diols. WPU-Cur particles showed higher absolute values of ζ potential compared with their nonloaded counterparts (Figure 4c), potentially due to the favorable interaction between curcumin and the EO segments,^53^ decreasing the distribution of EO segments at the micellar surface, resulting in an increased exposure of carboxylic acid groups on the surface.

The results from the fitting of SANS data of WPU-Cur samples are shown in Table 1 (data and fits in Figures S3 and S4). Since WPU in this research contains DMBA as the salt, we used a core–shell sphere form factor combined with the Hayter–Penfold structure factor for charged spheres to fit the SANS results and evaluate the possible effect of the triblock copolymer in the WPU drug loading system.^78,79^ The concentration of samples was set to 1 wt %. For the nonloaded samples, PEP35-E showed a smaller particle size (including core and shell), which is consistent with the trend observed in DLS, when compared to EPE40-E. The difference of size in PEP35-E and EPE40-E is due to the swelling caused by the hydrophilic segments (poly(ethylene oxide)) in the copolymer. Moreover, the arrangement of the amphiphilic diols is the major factor that impacts the swelling of micelles. The mobility of poly(ethylene oxide) in PEP35-E is much limited by propylene oxide and leads to a denser packing of hydrophilic segments in the core of micelles. The lower swelling degree of PEP35-E (∼3.8%) can also be confirmed by the volume fraction of micelles in SANS results. The hydrophilic tail groups in EPE40-E tended to be distributed on the surface of the micelles and relaxed in the solvent, which resulted in a thicker shell. By changing the ratio of hydrogen and deuterium in the solvent, and thus the neutron scattering length density (SLD), the SLD of the core in PEP35-E decreased with higher D_2_O concentration, which implies that water was more absorbed in the core of micelles. EPE40-E showed a different trend, that is, the SLD of the core remained constant even with a higher D_2_O concentration, but the SLD of the shell changed drastically, implying the solvent fraction in the shell is much more than that in PEP35-E. Compared to EPE40-E, PEP35-E showed a thinner thickness of the shell, which also indicated that the structure of the micelle is highly affected by water.

**Table 1:** Fitted SANS Results of Nonloaded and Loaded WPU Samples with Different Arrangements of Triblock Copolymers based on the Core–Shell Model

sample	PEP35-E			EPE40-E		
D–H ratio	100/0	80/20	60/40	100/0	80/20	60/40
radius (Å)	41 ± 1	41 ± 1	41 ± 1	51 ± 1	51 ± 1	51 ± 1
thickness (Å)	43 ± 1	43 ± 1	43 ± 1	110 ± 1	110 ± 1	110 ± 1
SLD core (10^–6^ Å^–2^)	1.260 ± 0.004	1.064 ± 0.007	1.103 ± 0.013	1.112 ± 0.004	1.107 ± 0.005	1.008 ± 0.006
SLD shell (10^–6^ Å^–2^)	6.010 ± 0.005	4.691 ± 0.009	3.42 ± 0.017	6.392 ± 0.001	4.953 ± 0.001	3.576 ± 0.001
SLD solvent (10^–6^ Å^–2^)	6.4000	4.9598	3.5796	6.4000	4.9600	3.5796
volume fraction	0.038 ± 0.0009	0.036 ± 0.0009	0.039 ± 0.0009	0.098 ± 0.0013	0.1047 ± 0.0013	0.1223 ± 0.0011
charge (e)	14.2 ± 0.1	20.4 ± 0.4	13.9 ± 0.3	7.8 ± 0.2	7.6 ± 0.3	7.6 ± 0.6
dielectric constant	70 ± 1	71 ± 8	71 ± 3	71 ± 4	71 ± 7	74 ± 12

	PEP35-Cur			EPE40-Cur		
D–H ratio	100/0	80/20	60/40	100/0	80/20	60/40
radius (Å)	36 ± 1	36 ± 1	36 ± 1	79 ± 2	79 ± 2	79 ± 2
thickness (Å)	50 ± 3	50 ± 3	49 ± 3	138 ± 2	138 ± 2	138 ± 1
SLD core (10^–6^ Å^–2^)	1.02 ± 0.004	1.040 ± 0.005	1.111 ± 0.006	1.278 ± 0.005	1.201 ± 0.003	1.129 ± 0.003
SLD shell (10^–6^ Å^–2^)	6.29 ± 0.002	4.896 ± 0.003	3.379 ± 0.015	6.131 ± 0.001	4.749 ± 0.001	3.430 ± 0.001
SLD solvent (10^–6^ Å^–2^)	6.4000	4.9598	3.5796	6.4000	4.9598	3.5796
volume fraction	0.028 ± 0.0003	0.028 ± 0.0003	0.028 ± 0.0003	0.0730 ± 0.0003	0.0730 ± 0.0003	0.0730 ± 0.0002
charge (e)	26.3 ± 0.3	25.2 ± 0.4	26.6 ± 0.9	27.0 ± 0.7	26.5 ± 1.2	22.5 ± 2.2
dielectric constant	90 ± 9	76 ± 10	56 ± 11	72 ± 3	72 ± 4	73 ± 7

For the curcumin-loaded samples, the radius of the core and shell showed the same trend as for the nonloaded samples. According to a previous study,^53^ both poly(propylene oxide) and poly(ethylene oxide) are able to interact with curcumin based on the simulation results. EPE40-Cur showed a lower SLD in the shell when compared to EPE40-E, which indicates that less water was incorporated in the shell and implied that curcumin was located in the hydrophilic shell. The involvement of curcumin also introduced the hydrophobic segments into the core of micelles, which resulted in the change of the SLD of the core with increasing D_2_O concentration. In PEP35-Cur, more hydrophilic segments were packed in the core, and curcumin can be well loaded and stabilized. The fitted SANS data of PEP35-Cur showed a smaller change of SLD in the core, and the SLD of the shell was closer to the SLD of the solvent, which indicated that the hydrophilic segments were more distributed in the core and interacted with curcumin, enhancing the denser packing of curcumin in the polymer.

### Curcumin Stabilization in Aqueous Solution by WPU-Cur

Figure 4d evaluates the ability of WPU to protect curcumin against degradation. The presence of curcumin was measured over time for WPU-loaded curcumin particles incubated in PBS at room temperature and compared with a control solution of free curcumin. Curcumin content in the control sample decreased by 59% in 21 days, due to environmental degradation. Encapsulated curcumin in EPE40-Cur and PEP35-Cur, tested under the same conditions, showed a decrease in curcumin content at the beginning, potentially due to the degradation of nonencapsulated curcumin in solution, and a stable curcumin content over 21 days of 75.28 ± 1.11 and 73.26 ± 1.62% for EPE40-Cur and PEP20-Cur, respectively. The stable curcumin content evidenced that curcumin was successfully protected from environmental degradation by the WPU emulsion in aqueous solutions.

### Curcumin Release Kinetics from WPU-Cur Films

Figure 4e and Table 2 present the in vitro curcumin release kinetics from the EPE- and PEP-based WPU formulations. The drug release curve was further analyzed by the Gallagher–Corrigan model^59,60^ which described a two-stage drug release process of polymeric carriers: (1) stage 1: bursting stage; and (2) stage 2: decomposing stage (Table 2). EPE40-Cur showed a higher rate of drug release when compared with PEP35-Cur. According to the fitted results of EPE40-Cur, 67.20% of the released curcumin was attributed to the bursting stage, while PEP35-Cur showed only 31.84%. In EPE-based WPU, the methyl side groups of PPO were distributed densely in the hydrophobic core, and PEO groups were distributed mainly in the corona phase. On the other hand, in PEP-based WPU, the hydrophobic PPO segments restricted the movement of PEO segments, decreasing their localization on the surface of the micelles,^24^ leading to a higher distribution of PEO segments in the core of PEP-based WPU micelles. Previous simulations show that both PEO and PPO segments have significant contributions in the interaction with the hydrophobic and hydrophilic sites of curcumin for the stabilization of curcumin in amphiphilic triblock copolymers.^80^ The higher PEO content in the PPO-rich micelle core of PEP35-Cur may have provided a more optimal local environment for the affinity toward curcumin, resulting in a higher retention of curcumin that survived the burst release stage.

**Table 2:** Fitted Drug Release Results of EPE40-Cur and PEP35-Cur

	*f*_b_	*k*_1_	*k*_2_	*f*_tmax_	*t*_2max_
EPE40-Cur	67.20	0.032	0.015	94.73	251.9
PEP35-Cur	31.84	0.036	0.013	81.59	192.6

**Figure 5:**
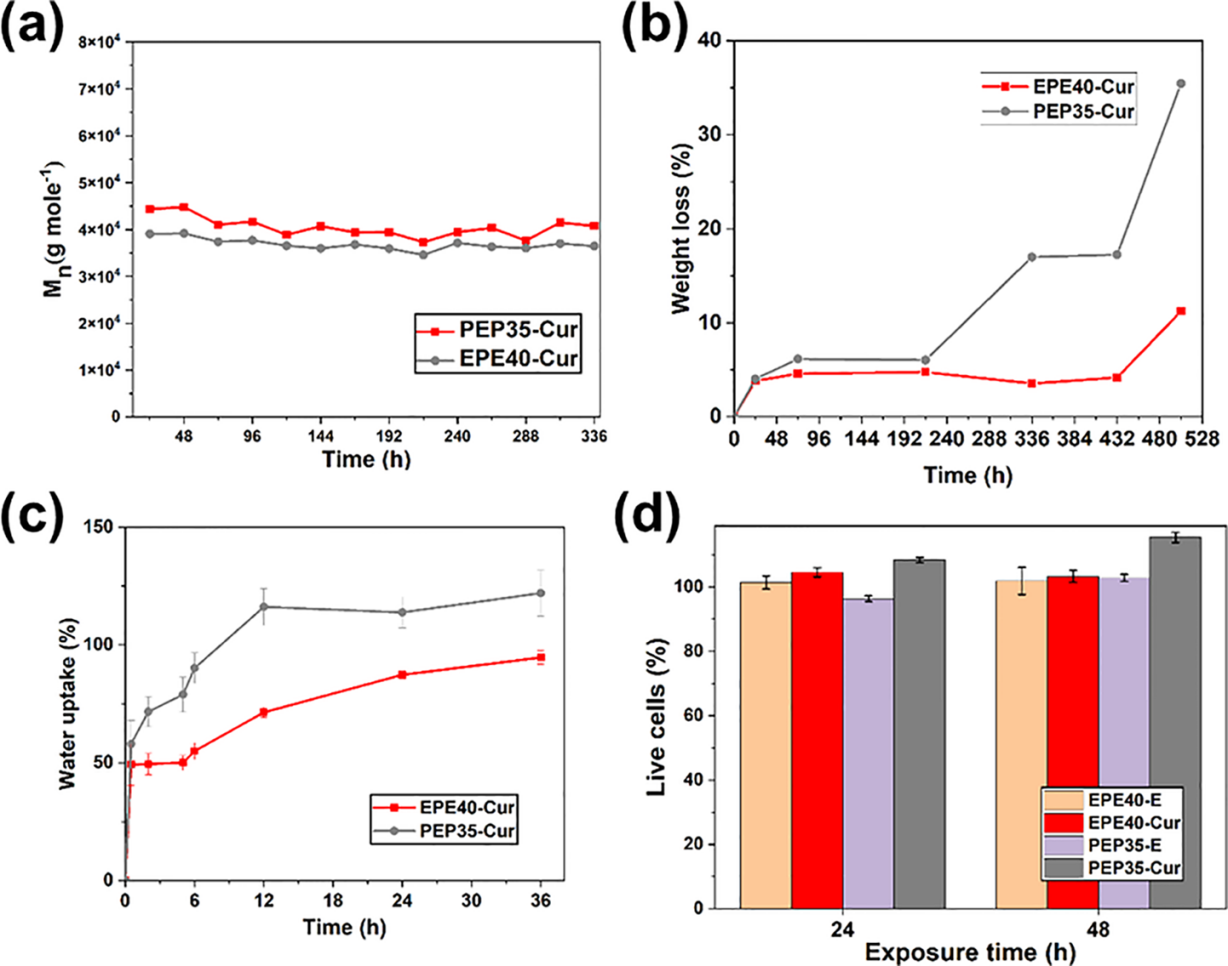
Drug release mechanistic studies and biocompatibility of WPU-Cur. (a) Weight loss of WPU-Cur during the incubation. (b) Weight loss of WPU-Cur during the incubation (PBS, 0.5% Tween-80, 37 °C). (c) Water uptake of PEP35-Cur and EPE40-Cur during the incubation. (d) Cytotoxicity assessment of WPU-E and WPU-Cur toward A549 cells (mean ± SD, *N* = 2).

The second stage following burst release was the decomposing stage,^59,60^ through which 49.75% of curcumin was released from PEP35-Cur and 27.53% from EPE40-Cur. To understand whether the release was due to chemical decomposition or physical decomposition, the molecular weight of curcumin-loaded WPU under the condition of the drug release test was tested by GPC (Figure 5a). Both EPE40-Cur and PEP35-Cur showed no significant changes in Mn, indicating that WPU-Cur was not chemically decomposed during incubation, suggesting that physical disintegration was the curcumin release mechanism at the second stage. The disintegration of the polymer films was quantified by the weight loss upon water incubation. Figure 5b demonstrates that PEP35-Cur had a higher weight loss upon water incubation, and the weight loss gradually increased at around 200 h, which is very close to *t*_2max_, the time of maximum release rate at the second stage of curcumin release. The water uptake of the WPU-Cur films was also recorded (Figure 5c). PEP35-Cur exhibited a higher water uptake when compared with EPE40-Cur after a 3h incubation, which continued to increase until the 36 h time point. These results suggest that the PEO segments in the core of the PEP35-Cur particles facilitated particle disintegration by their hydrophilicity and provided a more sustained curcumin release. Figure 5 shows that the drug release mechanism is highly dependent on the distribution of hydrophobic and hydrophilic segments of WPU, suggesting that through controlling the distribution of hydrophobic and hydrophilic segments, the drug release profile may be tuned based on the self-assembled structure and the drug–polymer interaction.

### Biocompatibility and Antibacterial Properties of WPU-Cur

One potential application of WPU-Cur films is wound dressing. For such an application, the biocompatibility and antibacterial properties are essential to the effectiveness of the system; such properties were also investigated in this study. Figure 5d evaluates the biocompatibility of WPU-E and WPU-Cur films toward epithelial cells (A549 cell), after 24 and 48 h of incubation using the MTT assay. No cytotoxicity was observed. Table 3 presents the antibacterial properties of EPE40-Cur and PEP35-Cur toward *E. coli*, compared with the blank polymer films, EPE40-E and PEP35-E. Data were analyzed according to the ASTM E2419 protocol, including the use of *N* = 3 and a standard deviation <0.21 to determine robust results. A decrease in viability was observed, and the inhibition rate resulted in 90.0% for EPE40-Cur and 50.6% for PEP35-Cur after 24 h of incubation for the loaded samples, compared with the controls. The higher inhabitation rate of EPE40-Cur may be attributed to its higher curcumin release rate during the first stage. Taken together, the findings evidence that EPE40-Cur and PEP35-Cur possess desirable characteristics for therapeutic applications such as wound dressing.

**Table 3:** Antibacterial Effectiveness of WPU-Cur

	microbiological load (CFU/mL)^a^		
	initial	after 24 h	inhabitation rate^a^
EPE40-E	5.2 × 10^5^	6.4 × 10^7^	
EPE40-Cur	5.2 × 10^5^	6.4 × 10^6^	90.0%
PEP35-E	4.1 × 10^5^	7.1× 10^7^	
PEP35-Cur	4.1 × 10^5^	1.6 × 10^8^	56.0%

a Results were analyzed according to ASTM E2419, *N* = 3, SD <0.21.

## Conclusions

Waterborne polyurethane based on amphiphilic triblock diols copolymerized with acrylates was successfully synthesized. This study shows that the utilization of EPE diols promotes the postcuring efficiency of WPU, which effectively enhances the mechanical strength of WPU films. Structural analysis shows that the enhancement in mechanical properties was due to the self-assembled micellar structure, induced by the polymer’s arrangement of hydrophilic–hydrophobic segments. To demonstrate that the control of micellar structure is key in control-release applications, curcumin was loaded into WPU, and the effect of the micellar structure on the drug loading and release profile was investigated. Curcumin-loaded WPU demonstrated a high loading efficiency and successfully stabilized curcumin from degrading under aqueous condition. The EPE diol-based WPU showed a more rapid release rate and higher inhabitation rate of bacterial growth. Finally, the curcumin-loaded WPU was biocompatible toward epithelial cells. Overall, these results revealed a combination of interesting properties of WPU based on triblock amphiphilic diols that can pave the way to a wide range of applications, such as in the coating industry as well as the biomedical field.

## Supporting Information Available

Fitting curves obtained from SANS analysis and detailed properties of WPU (PDF)
