# Supplementary material for: Structure Property Relationship of Micellar Waterborne Poly(Urethane-Urea): Tunable Mechanical Properties and Controlled Release Profiles with Amphiphilic Triblock Copolymers
Source: Langmuir. 2023 Jul 11;39(29):10033–46. doi: 10.1021/acs.langmuir.3c00921 (PMC10373496; doi:10.1021/acs.langmuir.3c00921)
Supplement: Supplementary file 1 — la3c00921_si_001.pdf [file la3c00921_si_001.pdf]

# *Supplementary Information*

# Structure property relationship of micellar waterborne poly(urethane-urea): tunable mechanical properties and controlled release profiles with amphiphilic tri-block copolymers.

*Shu-Yi Chen[1,2,3], Ida Kokalari[1], Steven R. Parnell[4], Gregory N. Smith[5], Bing-Hong Zeng[2,3], Tun-Fun Way[2,3], Fu-Sheng Chuang[2,3,6], Alina Y Rwei[1,*]*

AUTHOR ADDRESS

[1]Department of Chemical Engineering, Delft University of Technology, 2629 HZ, Delft, the Netherlands.

[2]Institute of Organic and Polymeric Materials, National Taipei University of Technology, 10608, Taipei, Taiwan.

[3]Research and Development Center for Smart Textile Technology, National Taipei University of Technology, 10608, Taipei, Taiwan.

[4]Department of Radiation Science and Technology, Delft University of Technology, 2629 HZ, Delft, the Netherlands.

[5]ISIS Neutron and Muon Source, Oxfordshire, OX11 0QX. UK.

[6]Department of Fashion and Design, Lee-Ming Institute of Technology, No. 22, Sec. 3, Tai-Lin Rd., Taishan Dist., New Taipei City 243, Taiwan

[*] Corresponding author. Email: A.Y.Rwei@tudelft.nl (A.Y.R.)

## Table of Contents

Table S1. Composition of WPU-H and WPU-E ........ S2

Table S2. Composition of WPU-Cur ........ S2

Figure S1. FT-IR spectra of WPU prepolymer and EPE20-H7 ........ S3

Figure S2. Zeta potential analysis of WPU-E ........ S4

Table S3. The ζ potential, molecular weight, and glass transform temperature (Tg) of WPU-E, WPU-H, and WPU-Cur ..... S5

Table S4. Molecular weight of WPU-Cur samples during the incubation in PBS, 0.5% Tween-80 (pH 7.4) ........ S6

Figure S3. The fitted SANS results of non-loaded WPU samples with different D2O concentration ........ S7

Figure S4. The fitted SANS results of curcumin loaded WPU samples with different D2O concentration ........ S8

Number of pages: 8

Number of figures: 4
Number of tables: 4

**Table S1. Composition of WPU-H and WPU-E**

| (mole) | HMDI | DMBA | Diol | TEA | EDA | HEMA | HEMA (wt%) |
|---|---|---|---|---|---|---|---|
| EPE20-H3 | 1.300 | 0.381 | 0.619 | 0.419 | - | 0.540 | 3.60 |
| EPE20-H7 | 1.645 | 0.394 | 0.606 | 0.433 | - | 1.161 | 7.20 |
| PEP25-H3 | 1.286 | 0.363 | 0.637 | 0.399 | - | 0.515 | 3.60 |
| PEP25-H7 | 1.614 | 0.375 | 0.625 | 0.413 | - | 1.105 | 7.20 |
| PPG-H7 | 1.582 | 0.356 | 0.644 | 0.391 | - | 1.048 | 7.20 |
| EPE40-H7 | 1.614 | 0.375 | 0.625 | 0.413 | - | 1.105 | 7.20 |
| PEP35-H7 | 1.582 | 0.356 | 0.644 | 0.391 | - | 1.048 | 7.20 |
| EPE20-E | 1.300 | 0.381 | 0.619 | 0.419 | 0.270 | - | - |
| EPE40-E | 1.285 | 0.363 | 0.637 | 0.399 | 0.257 | - | - |
| PEP25-E | 1.285 | 0.363 | 0.637 | 0.399 | 0.257 | - | - |
| PEP35-E | 1.270 | 0.344 | 0.656 | 0.378 | 0.243 | - | - |

**Table S2. Composition of WPU-Cur**

| (mole) | HMDI | Diol-40 | Diol-35 | DMBA | EDA | TEA | Curcumin(wt%) |
|---|---|---|---|---|---|---|---|
| EPE40-Cur | 1.600 | 0.550 | - | 0.450 | 0.600 | 0.450 | 4.55 |

| | | | | | | | |
|---|---|---|---|---|---|---|---|
| PEP35-Cur | 1.600 | - | 0.560 | 0.440 | 0.600 | 0.440 | 4.55 |

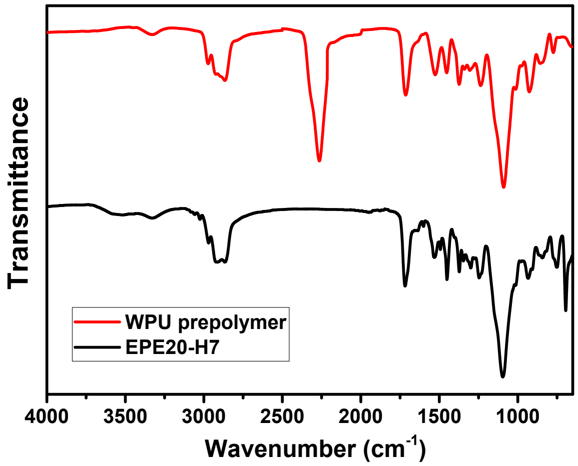


Figure S1. FT-IR spectra of WPU prepolymer and EPE20-H7

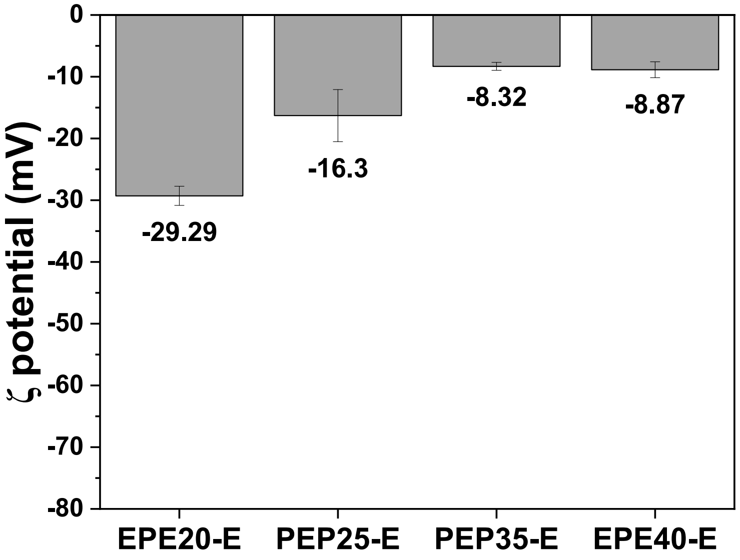


Figure S2. Zeta potential analysis of WPU-E

**Table S3. The ζ potential, molecular weight, and glass transform temperature ($T_g$) of WPU-E, WPU-H, and WPU-Cur**

| Sample | ζ potential (mV) | $M_n$ (g/ mole) | PDI | Tg (˚C) |
|---|---|---|---|---|
| EPE20-E | -29.29±1.55 | 53700 | 1.721 | -60.3 |
| PEP25-E | -16.30±4.22 | 28600 | 1.546 | -57.3 |
| PEP35-E | -8.32±0.64 | 30800 | 1.630 | -57.5 |
| EPE40-E | -8.87±1.29 | 55700 | 2.027 | -58.8 |
| EPE20-H3 | -26.89±2.44 | 33400 | 1.833 | -58.5 |
| EPE20-H7 | -23.93±1.15 | 24300 | 1.715 | -57.4 |
| PEP25-H3 | -16.95±1.09 | 24500 | 1.535 | -57.2 |
| PEP25-H7 | -33.21±3.44 | 20800 | 1.435 | -54.1 |
| EPE40-H7 | -17.87±0.86 | 37700 | 1.554 | -54.8 |
| PEP35-H7 | -7.80±0.54 | 48000 | 1.216 | -54.4 |
| PPG-H7 | -72.57±0.25 | 21200 | 1.407 | -54.9 |
| PEP35-Cur | -36.58±2.55 | 57700 | 2.47 | -45.0 |
| EPE40-Cur | -38.39±3.59 | 55500 | 2.23 | -56.2 |

Table S4. Molecular weight of WPU-Cur samples during the incubation in PBS, 0.5% Tween-80 (pH 7.4)

| Time (hr) | Sample | $M_n$ (g mole$^{-1}$) | $M_w$ (g mole$^{-1}$) | Sample | $M_n$ (g mole$^{-1}$) | $M_w$ (g mole$^{-1}$) |
|---|---|---|---|---|---|---|
| 24 | PEP35-Cur | 44,400 | 86,500 | EPE40-Cur | 39,000 | 79,000 |
| 48 | | 44,800 | 83,000 | | 39,200 | 81,500 |
| 72 | | 41,000 | 80,800 | | 37,400 | 81,100 |
| 96 | | 41,700 | 88,900 | | 37,700 | 82,000 |
| 120 | | 38,900 | 83,300 | | 36,500 | 76,300 |
| 144 | | 40,700 | 86,200 | | 36,000 | 83,600 |
| 168 | | 39,400 | 84,300 | | 36,800 | 74,400 |
| 192 | | 39,400 | 82,500 | | 36,000 | 71,900 |
| 216 | | 37,300 | 74,200 | | 34,600 | 72,300 |
| 240 | | 39,500 | 83,600 | | 37,100 | 80,000 |
| 264 | | 40,400 | 85,700 | | 36,300 | 80,900 |
| 288 | | 37,700 | 80,700 | | 36,000 | 84,400 |
| 312 | | 41,500 | 77,700 | | 37,000 | 87,800 |
| 336 | | 40,800 | 82,700 | | 36,500 | 79,000 |

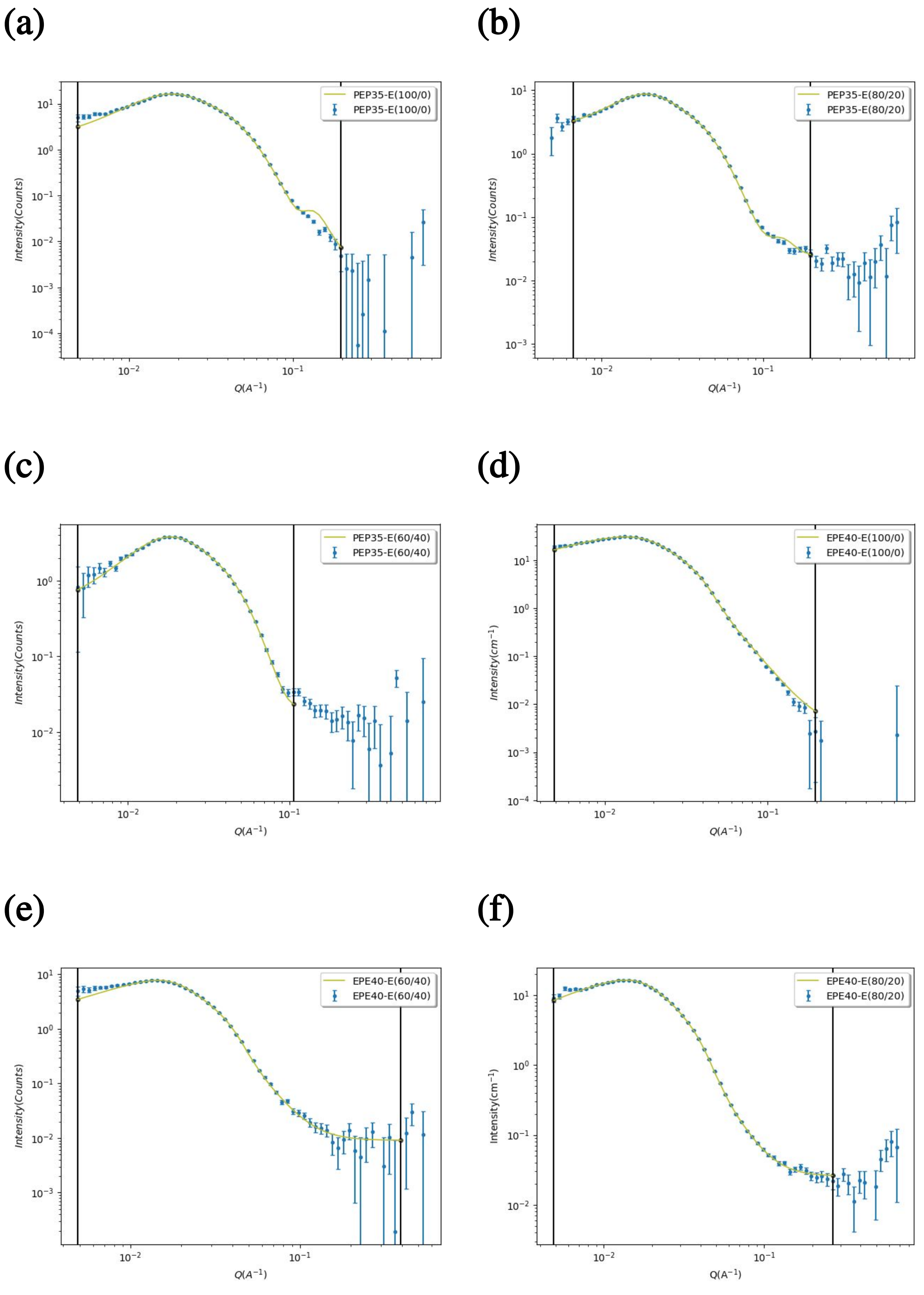


Figure S3. The fitted SANS results of non-loaded WPU samples with different $D_2O$ concentration

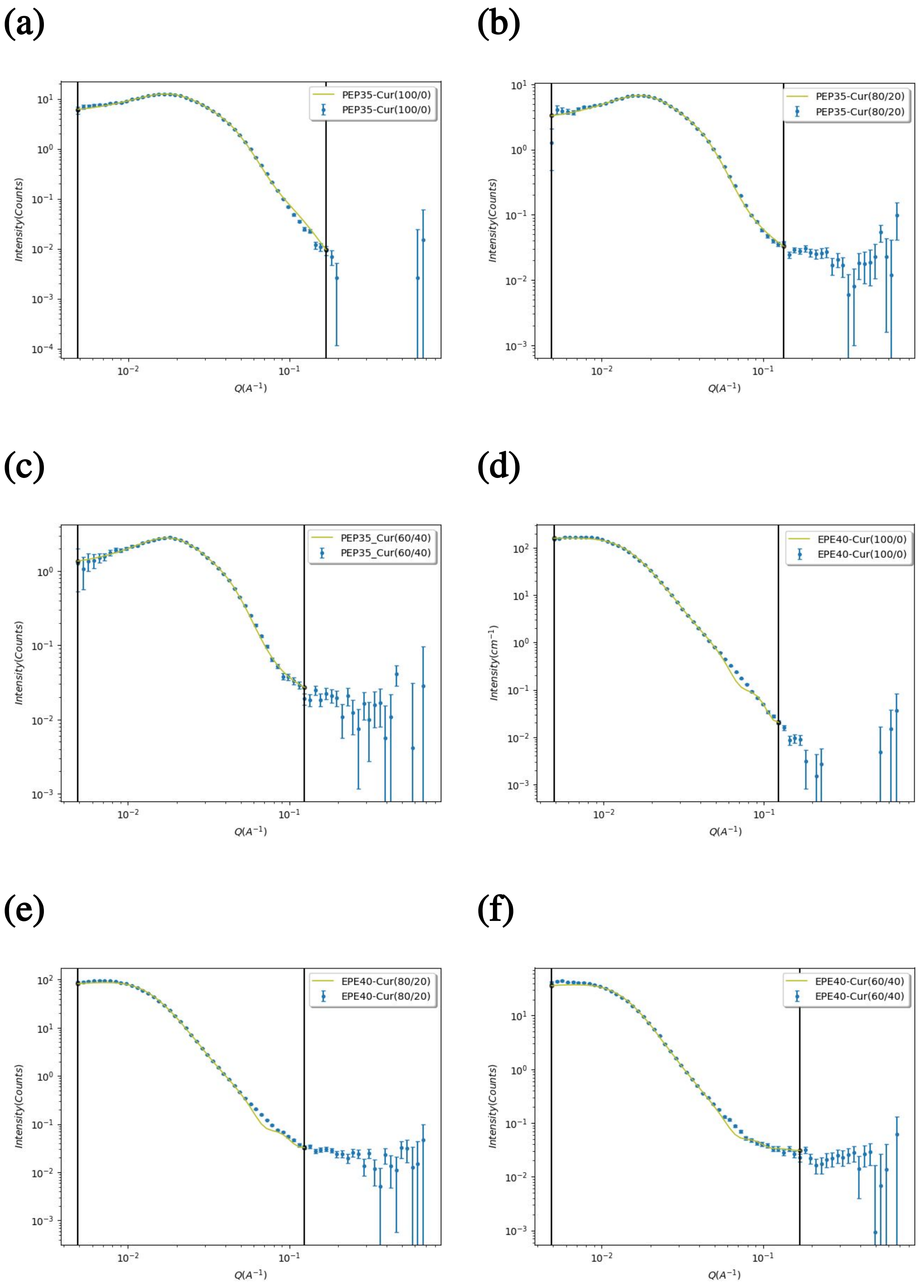


Figure S4. The fitted SANS results of curcumin loaded WPU samples with different $D_2O$ concentration
